# Arthroscopic rotator interval release for frozen shoulder, comparative study between diabetic and non-diabetic patients

**DOI:** 10.1051/sicotj/2022036

**Published:** 2022-08-19

**Authors:** Wessam Tawfeek, Ahmad Addosooki, Moustafa Elsayed

**Affiliations:** Orthopaedic Department, Sohag University Hospital 82524 Sohag Egypt

**Keywords:** Arthroscopic, Release, Rotator interval, Frozen shoulder

## Abstract

*Introduction:* The objective of this study is to evaluate the outcomes of arthroscopic rotator interval release for the treatment of frozen shoulder and compare the results in patients with and without diabetes. *Methods*: thirty-two patients with frozen shoulders were divided into two groups; 19 diabetics and 13 non-diabetics. All patients underwent arthroscopic rotator interval release. The VAS and UCLA score were assessed pre-operatively and post-operatively; after 1, 3, and 12 months and compared between groups. *Results*: The VAS and UCLA score was significantly improved in both groups during follow-up intervals (*p* < 0.01). There was no significant difference between diabetic and non-diabetic patients in VAS and UCLA scores during follow-up times (*p*-values > 0.05). *Conclusion*: Arthroscopic rotator interval release provides significant improvement of frozen shoulder with no difference in results between diabetic and non-diabetic patients.

Level of evidence: Level 2; Prospective Comparative study.

## Introduction

Frozen shoulder (FS), initially defined by Codman in 1934, is the third most common cause of musculoskeletal pain [1–3]. It is classically presented by pain and limitation of active and passive shoulder movement [4–6]. The common age affected is between 40 and 60 years [2, 3, 7]. The non-dominant side is more often affected [6], 6–17% of patients have bilateral involvement, and the incidence is slightly higher in females by a ratio of 1.4:1 [8].

Lundberg classified frozen shoulder into two types; idiopathic (primary) and secondary. The pathology of the idiopathic type remains unclear. Secondary frozen shoulder is described when the condition is associated with known comorbidities such as diabetes mellitus, the most common cause of trauma, cervical disease, and ischemic heart disease [3, 9].

A relation between diabetes and frozen shoulder has been confirmed [10, 11]. Its prevalence increases up to 20% in diabetic patients while it is about 2–5% in all population [2, 6], the incidence is slightly higher in Type 2 diabetes (about 29%) compared to Type 1 (about 10%) [10, 11].

The underlying pathology of frozen shoulder is still uncertain, but the contracture of the rotator interval (RI) and coracohumeral ligament (CHL) appears to be the main factor [12].

The main goal of treatment of frozen shoulder is to improve range of movement (ROM). Several options, including non-steroidal anti-inflammatory, corticosteroids, corticosteroid local injection, capsular hydrodistension, physiotherapy, mobilization under anesthesia, and arthroscopic or open surgery, were described with variable results [13, 14].

With the development of arthroscopic techniques, arthroscopic release is the treatment of choice in patients with frozen shoulders resistant to conservative treatment. It provides great improvement in the range of movement with a low complication rate [3–5].

The optimal technique of arthroscopic release remains a controversial issue. Some surgeons recommend subscapularis release with the standard anteroinferior release, while others also added posterior capsular release to improve internal rotation [15, 16].

Although the rotator interval is not the only part involved in the shoulder capsule, some authors have adopted that the rotator interval and CHL have a great effect on the pathogenesis of the frozen shoulder [5, 17, 18].

Several studies were conducted to evaluate and compare the results of arthroscopic capsular release (360° release) in diabetic and non-diabetic patients [19–23]. In this study, we released only RI and CHL, which we believe that contracture is the main pathology in frozen shoulder. To our knowledge, there are no previous studies used this technique before.

This study aimed to evaluate the efficacy of arthroscopic rotator interval and coracohumeral ligament release for frozen shoulder and compare the results between diabetic and non-diabetic patients.

## Materials and methods

It is a prospective study that included 32 patients who suffered from FS. This was diagnosed clinically as chronic shoulder pain with a limitation of both active and passive ROM. The patients were categorized into two groups; the diabetic group contained 19 patients (2 males and 17 females) with an average age of 47.47 years (37–61), and a non-diabetic group contained 13 patients (4 males and 9 females) with average age 50.15 years (39–60). Patients’ demographics of both groups are presented in Table 1.

**Table 1:** Demographic data of the patients.

	Diabetics (*n* = 19)	Non-diabetics (*n* = 13)	*P*-value
Age			
Range	37–61	39–60	0.224
Mean ± SD	47.47 ± 6.16	50.15 ± 5.74	
Gender			
Male	2 (10.5%)	4 (30.8%)	0.150
Female	17 (89.5%)	9 (69.2%)	
Side affected			
Left	11 (57.9%)	9 (69.2%)	0.515
Right	8 (42.1%)	4 (30.8%)	
Hand			
Dominant	8 (42.1%)	3 (23.1%)	0.266
Non-dominant	11 (57.9%)	10 (76.9%)	

Chi-square test.

* Statistically significant difference (*p* < 0.05).

** Highly statistically significant difference (*p* < 0.01).

Inclusion criteria were; symptomatic frozen shoulder affecting patient’s daily activities and failed to respond to conservative therapy including NSAIDs, physiotherapy, and local steroids injection for at least 3 months.

We excluded from this study; patients with glenohumeral osteoarthritis, rotator cuff tears, history of fracture of the proximal humerus, history of operative intervention in the affected shoulder, bilateral FS, and patients who underwent mobilization under anesthesia or hydrodistention.

All patients were subjected to careful evaluation regarding history, clinical examination, and radiographic evaluation using standard plain radiographs and MRI to confirm the diagnosis and rule out any intra-articular pathology. The study was approved by the ethical committee of our institution, and informed consent was taken from all patients.

All patients underwent arthroscopic rotator interval and coracohumeral release by the same surgical team. Clinical evaluation was done by Visual Analog Scale (VAS) and UCLA (University of California Los Angeles) score. The assessment was done pre-operatively and post-operatively after one, three, and twelve months.

### Surgical technique

The procedure was done under general anesthesia in a beach chair position. The patient was reexamined under anesthesia to check ROM.

The posterior portal was determined to be approximately 2 cm medial and inferior to the posterolateral angle of the acromion. The shoulder joint was distended with about 20–30 cc of saline then the arthroscopy was introduced through the posterior portal. An anterior portal was established under arthroscopic visualization between the biceps tendon superiorly and subscapularis tendon inferiorly. Synovectomy was done using an arthroscopic shaver.

**Figure. 1:**
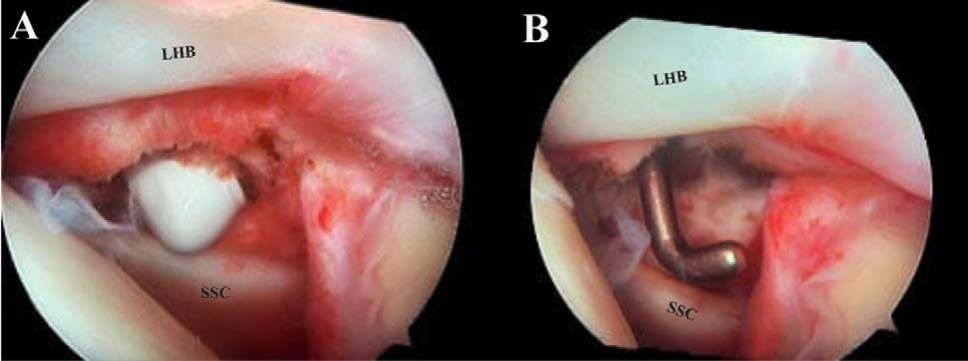
Intraoperative Arthroscopic picture of left shoulder (viewed from posterior portal) showing the rotator interval between long head of biceps (LHD) superiorly and subscapularis tendon (SSC) inferiorly. A. A radiofrequency ablator introduced via the anterior portal and releases all the layers of RI. B. A probe is used to check completion of release.

RI release with CHL release was performed using a radiofrequency ablation device up to the base of the coracoid (Figure 1). The range of motion was checked again after the procedure was completed.

### Post-operative Protocol

A supervised physiotherapy program was applied to all patients immediately post-operative. An arm sling was applied for one week. During which scapular and pendular exercises were started. After one week, the sling was removed, and passive movement and capsular stretching exercises were introduced. Active-assisted and Active movement exercises were initiated after two weeks. Strengthening exercises were started by the end of the 4th week.

### Statistical analysis

At first, the data were tested for homogeneity variances and normality by the Kolmogorov–Smirnov test. Categorical variables were presented by number and percent (*N*, %), while continuous variables were presented by mean and standard deviation (Mean, SD). For comparing both groups, we used the fisher exact test and Chi-square test to compare categorical variables, while comparison between continuous variables was carried out by the Mann–Whitney test for non-parametric data (VAS and UCLA) and independent samples T-test for parametric data. Wilcoxon test was used to compare VAS and UCLA scores between pre-operative and post after one month, after three months, and after 12 months within the same group. *P* value < 0.05 was considered statistically significant. IBM SPSS 28.0 software was used for analysis.

## Results

There was no statistically significant difference between both groups as regards patients’ age (*p* = 0.224), sex (*p* = 0.150), the side affected (*p* = 0.515), and dominant hand (*p* = 0.266).

**Figure 2:**
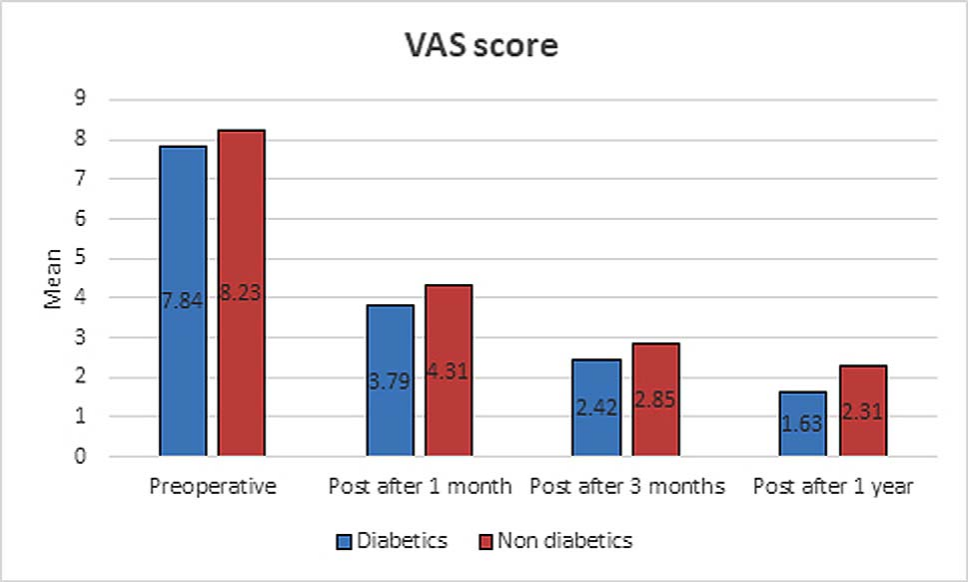
An illustrative diagram showing post-operative improvement of VAS in diabetic and non-diabetic patients after RI release (at 1, 3 and 12 months follow-up). No difference was found in VAS between both groups.

Both groups achieved significant improvement in VAS during follow-up intervals (*p* < 0.01), while there were no statistically significant differences in VAS between the two study groups during follow-up times (*p*-values > 0.05) (Table 2) (Figure 2).

**Table 2:** VAS score results of both groups.

VAS score	Diabetics (*n* = 19)	Non-diabetics (*n* = 13)	*P*-value
Pre-operative			
Range	6–9	6–10	0.279
Mean ± SD	7.84 ± 0.96	8.23 ± 1.09	
Post 1 month			
Range	2–6	3–6	0.175
Mean ± SD	3.79 ± 1.13	4.31 ± 0.85	
Post 3 month			
Range	1–5	1–4	0.251
Mean ± SD	2.42 ± 1.22	2.85 ± 0.99	
Post 1 year			
Range	0–5	0–5	0.175
Mean ± SD	1.63 ± 1.42	2.31 ± 1.32	
*P*1	<0.001**	<0.001**	
*P*2	<0.001**	<0.001**	
*P*3	<0.001**	<0.001**	

Mann–Whitney test; to compare the two groups each time.

Wilcoxon test; to compare between periods of time in each group.

*P*1: comparison between pre-operative and post 1 month for each group.

*P*2: comparison between post 1 month and post 3 months for each group.

*P*3: comparison between post 3 months and post 1 year for each group.

*Statistically significant difference (*p* < 0.05).

**Highly statistically significant difference (*p* < 0.01).

**Figure 3:**
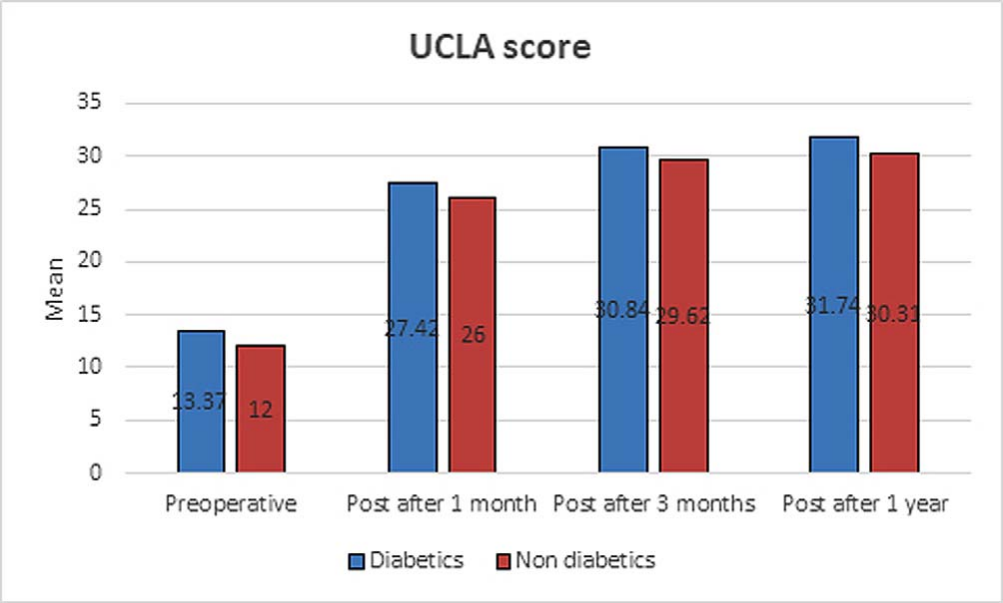
An illustrative diagram showing post-operative improvement of UCLA score in diabetic and non-diabetic patients after RI release (at 1, 3, and 12 months follow-up). No difference was found in UCLA score between both groups.

Both groups achieved significant improvement in UCLA scores during follow-up intervals (*p* < 0.01), and by comparing both groups, there were no statistically significant differences in UCLA scores during follow-up times (*p*-values > 0.05) (Table 3) (Figure 3). The mean operative time was 36.5 min, with no complications recorded.

**Table 3:** UCLA score results of both groups.

UCLA score	Diabetics (*n* = 19)	Non-diabetics (*n* = 13)	*P*-value
Pre-operative			
Range	8–18	7–18	0.304
Mean ± SD	13.37 ± 3.73	12 ± 3.19	
Post 1 month			
Range	20–32	20–30	0.117
Mean ± SD	27.42 ± 3.19	26 ± 2.97	
Post 3 month			
Range	24–34	25–33	0.145
Mean ± SD	30.84 ± 3.11	29.62 ± 2.43	
Post 1 year			
Range	22–35	20–35	0.126
Mean ± SD	31.74 ± 3.51	30.31 ± 3.61	
*P*1	<0.001**	<0.001**	
*P*2	<0.001**	<0.001**	
*P*3	<0.001**	0.002**	

Mann–Whitney test; to compare the two groups each time.

Wilcoxon test; to compare between periods of time in each group.

*P*1: comparison between pre-operative and post 1 month for each group.

*P*2: comparison between post 1 month and post 3 months for each group.

*P*3: comparison between post 3 months and post 1 year for each group.

*Statistically significant difference (*p* < 0.05).

**Highly statistically significant difference (*p* < 0.01).

## Discussion

Arthroscopic release in the frozen shoulder can produce significant and sustained relief when conservative treatment has failed [17, 24]. Some authors reported lower results in diabetic patients than non-diabetics [25], however, others indicated no difference between both groups [26]. In this study, we found a significant improvement in all patients, diabetics, and non-diabetics, after arthroscopic rotator interval release, and both groups showed similar satisfactory outcomes. Both diabetic and non-diabetic patients had a significantly lower VAS and higher UCLA score one month, three months, and one year post-operatively than pre-operative.

We have a few limitations in our study; the first is a small study population. The second is its non-randomized design. However, we have some points of strength, including that we prospectively selected the patients with strict inclusion criteria, all patients operated by the same team using the same technique, and both groups were comparable in demographic variables, despite the presence or absence of diabetes.

Frozen shoulder is one of the most common musculoskeletal disorders [2, 3]. It is diagnosed clinically as chronic shoulder pain and limitation of active and passive shoulder movement, especially internal rotation [4–6]. Usually, there are no specific radiological findings, but sometimes MRI shows thickening of the rotator interval in axial images and thickening of the axillary pouch in coronal images [5, 6].

Patients with diabetes may have worse outcomes from frozen shoulder; clinicians should monitor those patients and recommend further treatment if symptoms persist for a long time [10]. Diabetic patients also may have slower post-operative functional recovery after arthroscopic release, so they should be counseled clearly about the results of the procedure [20, 22].

Our results are similar to previous studies (Table 4) that compared the results of arthroscopic capsular release in diabetic and non-diabetic patients. Lyhne et al. [19], although they performed anterior and posterior capsular release. They concluded that all patients, whether diabetic or non-diabetic, equally improved after arthroscopic capsular release when operated on regardless of diabetic status.

**Table 4:** Review of literature of relevant studies that compared the results of arthroscopic release in diabetic and non-diabetic frozen shoulder patients.

_Study_	_No. of patients_	_Avg. age (years)_	_Follow up_	_Surgical technique_	_Functional score_	_Results_	_Encountered complications_
_Lyhne JM et al. (2019) [_ 19_]_	_18 diabetic 75 non-diabetic_	_Diabetic: 55.2 non-diabetic: 56_	_6 months_	_Arthroscopic capsular release (360° arthroscopic capsulotomy)._	_-Oxford Shoulder Score (OSS)._ _-Visual Quality Scale (VQS)._	_Significant improvement in both groups._ _No significant difference between both groups._	_No complications_
_Cho CH et al. (2016) [_ 20_]_	_17 diabetic 20 non-diabetic_	_55.6_	_48.4_ ± _15.8 months_	_Arthroscopic capsular release (360° arthroscopic capsulotomy)._	_VAS (Visual Analog Score)._ _UCLA (University of California Los Angeles score)._ _ASESS (American Shoulder and Elbow Surgeons score)._	_Significant improvement in both groups._ _No significant difference between both groups except ASESS was significantly lower in diabetics at 12 months._	_No complications_
_Cinar M et al. (2010) [_ 21_]_	_14 diabetic 12 idiopathic_	_50_	_48.5 months in diabetic 60.2 months in idiopathic_	_Arthroscopic capsular release (360° arthroscopic capsulotomy)._	_University of California, Los Angeles (UCLA)._ _Constant Scoring Systems._	_Significant improvement in both groups._ _UCLA scores: no significant difference._ _Constant scores: significantly lower in diabetics._	_No complications_
_Mehta SS et al. (2014) [_ 22_]_	_21 diabetic 21 non-diabetic_	_54.5_	_24 months_	_Arthroscopic capsular release (360° arthroscopic capsulotomy)._	_Modified Constant-Murrley score._	_- Significant improvement in both groups.- No significant difference between both groups at 2 yrs._	_No complications_
_Lei GY et al. (2019) [_ 23_]_	_32 diabetics 24 non-diabetic_	_Diabetic: 56.9 Non-Diabetic: 53.6_	_12 months_	_Arthroscopic capsular release (360° arthroscopic capsulotomy)._	_Visual analog scale (VAS)._ _Constant-Murley Shoulder Score (CMSS)_ _University of California at Los Angeles (UCLA) shoulder score._ _Oxford shoulder score (OSS)._	_Significant improvement in both groups._ _No significant difference between both groups except CMSS was significantly lower in diabetics._	_No complications_

Cho et al. [20] reported similar results to our study, where both diabetic and non-diabetic patients significantly improved in ROM and clinical scores. No significant differences between both groups in VAS and UCLA scores, while the mean American Shoulder and Elbow Surgeons (ASES) score in the diabetic group was significantly lower than the non-diabetic group 12 months post-operatively. The ASES score is a subjective questionnaire. It does not include objective parameters like the UCLA score we used, which contain both subjective and objective measurements.

Cinar et al. [21] reported that non-diabetic patients achieved significantly higher Constant scores after arthroscopic release than the diabetic group. They concluded that in frozen shoulder, arthroscopic capsular release in diabetic patients had lower results as regards Constant score and ROM. Both groups did not show a significant difference regarding the duration needed for pain relief and restoring ROM.

Mehta et al. [22] reported improvement in diabetics and non-diabetics in the modified constant score. However, the scores in non-diabetics were significantly higher than diabetics after six months of follow-up, although both groups significantly improved from the pre-operative scores. However, diabetic patients suffered more from persistent movement limitation for two years post-operatively.

Our results demonstrated significant success in clinical outcomes with arthroscopic RI release; it is a simple technique, less time-consuming, and safer compared to other capsular release techniques.

In Conclusion, arthroscopic RI release provides good clinical outcomes in the treatment of frozen shoulder and can be used effectively in diabetic and non-diabetic patients with no difference in results.
